# Trophic position, elemental ratios and nitrogen transfer in a planktonic host–parasite–consumer food chain including a fungal parasite

**DOI:** 10.1007/s00442-020-04721-w

**Published:** 2020-08-17

**Authors:** Virginia Sánchez Barranco, Marcel T. J. Van der Meer, Maiko Kagami, Silke Van den Wyngaert, Dedmer B. Van de Waal, Ellen Van Donk, Alena S. Gsell

**Affiliations:** 1 Copernicus Institute of Sustainable Development, University of Utrecht, Utrecht, The Netherlands; 2 Department of Marine Microbiology and Biogeochemistry, Royal Netherlands Institute for Sea Research (NIOZ) and Utrecht University, Utrecht, The Netherlands; 3 Graduate Schools of Environment and Information Sciences, Yokohama National University, Yokohama, Japan; 4 Department of Experimental Limnology, Leibniz-Institute of Freshwater Ecology and Inland Fisheries, Berlin, Germany; 5 Department of Aquatic Ecology, Netherlands Institute of Ecology (NIOO-KNAW), Wageningen, The Netherlands

**Keywords:** Chytrid, Mycoloop, Phytoplankton, Stable isotope analysis, Zooplankton

## Abstract

Parasitism is arguably the most commonly occurring consumer strategy. However, only a few food web studies assess how well stable isotopes reflect the trophic position of parasitic consumers and results are variable. Even fewer studies have measured the nutrient transfer by parasitic consumers, hindering an assessment of their role in nutrient transfer through food webs. Here we used a food chain consisting of a diatom as host, a chytrid as its parasitic consumer and a rotifer as the predatory consumer of the chytrid, to assess the trophic position of all three food-chain components using their natural ^13^C and ^15^N isotope signatures, and to measure the nitrogen transfer from the host via the chytrid to the rotifer by tracing ^15^N of a labelled host up the food chain. Additionally, we measured the carbon to nitrogen (C:N) ratios of all food-chain components. Natural isotope abundance results showed no clear ^15^N enrichment in the chytrid or rotifer relative to the primary producer. However, estimates of nitrogen transfer indicated that about 14% of host nitrogen was transferred per day from host to chytrid during infection epidemics and that some of this nitrogen was also transferred onward to the rotifer. Moreover, C:N ratios decreased with trophic level, suggesting that the chytrid provided a high-quality food source to the rotifer. In conclusion, our results support the “mycoloop”. The mycooloop proposes that chytrid infections allow the transfer of nutrients bound in large, inedible phytoplankton to zooplankton through the production of edible transmission spores, thereby rerouting nutrients back into the food web.

**Electronic supplementary material:**

The online version of this article (10.1007/s00442-020-04721-w) contains supplementary material, which is available to authorized users.

## Introduction

Food webs describe the transfer of energy and nutrients through ecosystems based on trophic interactions. A substantial portion of consumers in food webs is parasitic consumers of free-living organisms (Dobson et al. 2008; Kuris et al. 2008), but the occurrence and role of parasitic consumers have been neglected in most food web studies, including aquatic ones (Lafferty et al. 2008). Even though parasitic consumers dominate food web links (Lafferty et al. 2006), only few studies assess the trophic position of phytoplankton parasitic consumers and their role in nutrient transfer from host via parasitic consumer to zooplankton (Kagami et al. 2007). Chytrids, fungal parasitic consumers of phytoplankton, have been proposed to add previously unrecognized trophic links and to change the flow of carbon (C) in aquatic ecosystems (Grami et al. 2011; Kagami et al. 2014; Rasconi et al. 2014). Chytrids also play a role in regulating the dynamics and population densities of phytoplankton hosts and in the structure of pelagic plankton food webs (Ibelings et al. 2004). For instance, epidemics of parasitic chytrids on phytoplankton can increase host genetic diversity (Gsell et al. 2013), decrease uni-algal bloom size and duration, allowing competing species to thrive [see references in (Frenken et al. 2017a)] and thereby play a role in the seasonal succession of phytoplankton (Van Donk and Ringelberg 1983). Nevertheless, physical and chemical factors, together with grazing, have been considered to be the major controlling factor of phytoplankton populations, and most aquatic food web models still exclude parasitic consumers (Frenken et al. 2017a).

**Fig. 1:**
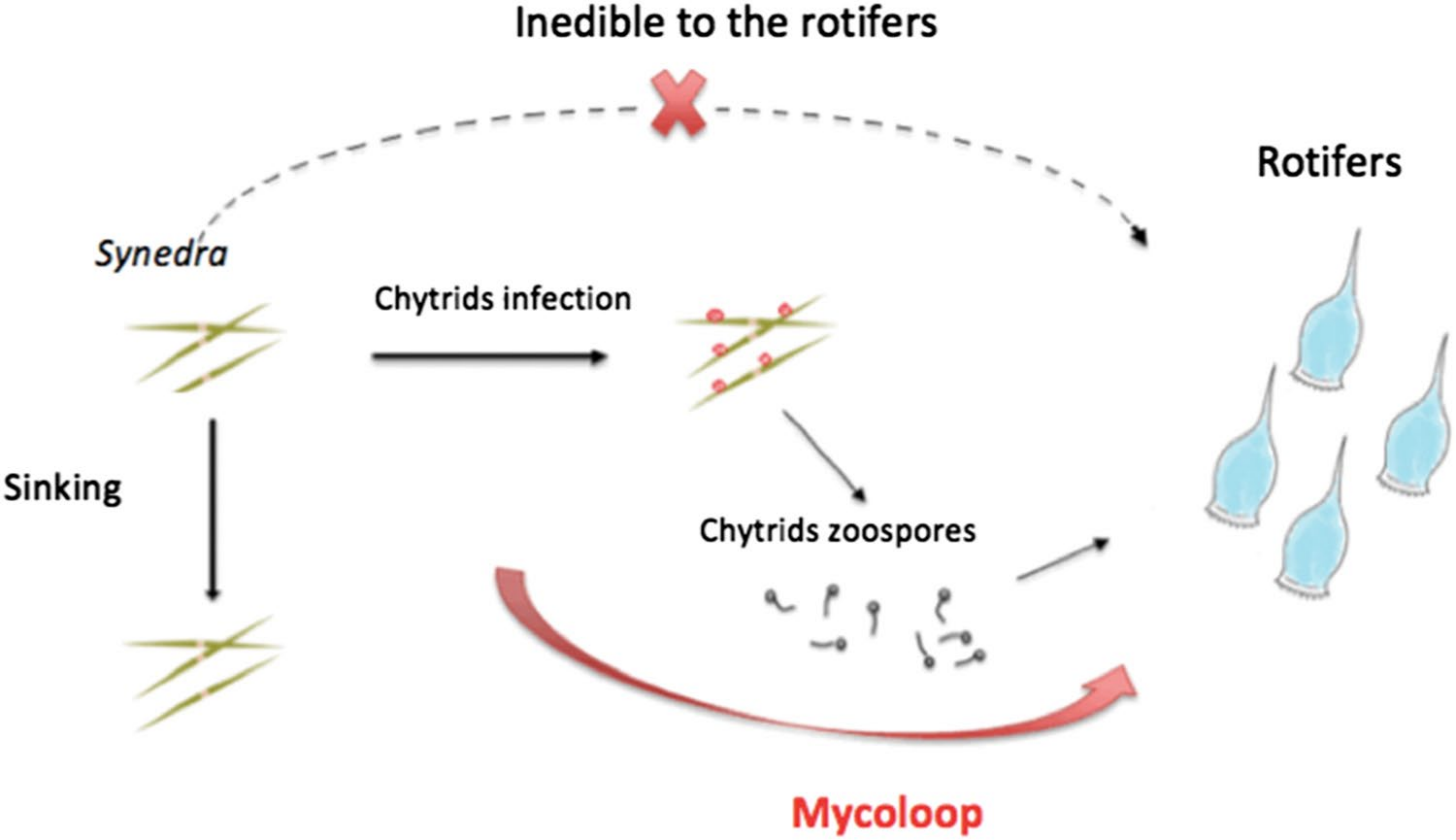
Diagram of the mycoloop. The food web system includes the inedible diatom (*Synedra*), the obligate parasitic consumer of the diatom (chytrid) with a sessile (sporangium) and a motile (zoospore) life stage, and the rotifer (*Keratella*), which can consume the chytrid zoospores but not the host diatom. While *Synedra* is inedible to *Keratella*, its nutrients may still be transferred to the rotifer via infection propagules (zoospores)

Chytrid infections of large phytoplankton have been suggested to be ecologically important for food web functioning. The “mycoloop” proposes that chytrid infections of phytoplankton transfer elements from large or inedible phytoplankton to zooplankton through the production of edible transmission propagules and thereby change the flow of nutrients through the food chain (Kagami et al. 2014). For example, large diatom species such as *Synedra* (25–400 μm) are inedible to common zooplankton species such as the rotifer *Keratella*, and the elements contained in the diatom cells were thought to be lost from the pelagic food web by sedimentation (Ibelings et al. 2004). However, chytrid fungi parasitizing these large diatoms take up nutrients from the host cells and produce free swimming zoospores, which in turn, can be eaten by zooplankton (Kagami et al. 2007), including rotifers such as *Keratella* (Frenken et al. 2018, 2020)*.* In this way, chytrids directly link inedible phytoplankton to zooplankton (Kagami et al. 2014) (Fig. 1). Excretion of organic matter from phytoplankton cells may be taken up by bacteria which then can be consumed by small-bodied zooplankton, leading to an alternative pathway for nutrient transfer from inedible phytoplankton to zooplankton (Shniukova and Zolotareva 2015). This leakage of organic matter may increase with chytrid infection, possibly leading to higher bacteria numbers that could intensify this route (Senga et al. 2018). Thus, the mycoloop may be an important route establishing or enhancing the flow of nutrients in food webs that include inedible phytoplankton and parasitic consumers (Frenken et al. 2018; Haraldsson et al. 2018). To the best of our knowledge, the actual amount of nutrient transfer via the mycoloop has not yet been measured.

The presence of chytrids may not only affect the quantity of food that is transferred from the inedible phytoplankton to zooplankton, but also its quality in terms of nutritional value. Zoospores of chytrids are assumed to provide a source of high-quality food to zooplankton as they are nutrient rich as shown by their relatively low carbon to nutrient ratio (Kagami et al. 2007; Frenken et al. 2017b), transfer host-derived essential lipids and synthesise sterols de novo (Gerphagnon et al. 2018). Yet, little is known about the ecological stoichiometry of the mycoloop, and only limited data is available about the actual amounts of carbon (C) or nitrogen (N) transferred from freshwater phytoplankton to chytrids (see Grami et al. 2011; Haraldsson et al. 2018; Rasconi et al. 2014 for carbon fluxes and Frenken et al. 2017a, b for a review).

Natural abundances of ^13^C and ^15^N isotopes can be used to analyse food web structure and to estimate contribution of different food sources (Layman et al. 2012). Nitrogen isotopic ratios (δ^15^N) show a gradual enrichment with trophic transfer (Minagawa and Wada 1984) and can be used to estimate the trophic position of organisms (Phillips et al. 2014). Carbon isotopic ratios (δ^13^C), on the other hand, stay relatively constant with trophic transfer and, therefore, inform about the food source of the consumer (Rounick and Winterbourn 1986; Post 2002). Consumers normally show an enrichment of approximately 3.4 ‰ in ^15^N and of 1 ‰ in ^13^C compared to their resource (Tilley et al. 2013). However, trophic fractionation (i.e. isotopic difference between consumer and resource) has been shown to vary considerably in laboratory and field studies with particularly variable outcomes for herbivores (Vander Zanden and Rasmussen 2001). Part of this variability has been associated with species identity, type of tissue analysed and the quantity or quality of the resource (Brauns et al. 2018). Trophic fractionation between parasitic consumers and their host shows large variability, where parasitic consumer ^13^C or ^15^N may either become depleted, remain unaltered or become enriched relative to its host (see Sabadel et al. 2019 for a review), rendering the question of the trophic position of parasitic consumers based on isotopic signatures inconclusive. Additionally, stable isotopes can be used for labelling studies or “stable isotope probing” studies (SIP) (Fry 2006). By enriching organisms with known amounts of heavy isotopes, ^13^C and/or ^15^N, the transfer of this label can be traced along trophic interactions to quantify the flux of nutrients from one trophic level to the next (Fry 2006). Hence, stable isotope analysis offers opportunities for improving our understanding of the role of chytrids in transferring nutrients and its trophic position in freshwater food chains.

Here, we assessed the trophic position of chytrids based on isotopic signatures and the transfer of N using a trophic chain of the diatom host *Synedra* sp., its chytrid parasitic consumer * Zygophlyctis planktonica*, and the rotifer *Keratella cochlearis*. These three species form a linear food chain as the rotifer can consume the zoospores of the chytrid (Frenken et al. 2020) but not the relatively large diatom host. First, to test whether stable isotope signatures reflected the trophic positions of all food chain components, natural abundances of ^15^N and ^13^C were assessed in host, infected host, chytrid zoospores and in rotifers exposed to a diet of either uninfected *Synedra* (inedible) or infected *Synedra* (producing edible zoospores). Second, to assess N-transfer through the food chain, the same experimental set up was repeated using ^15^N-enriched host to trace the flow of ^15^N from host to chytrid zoospores (quantitatively) and from uninfected or infected diatom host cultures to rotifers (qualitatively). Concomitantly, we assessed the C:N elemental ratios of all food chain components to evaluate changes in their nutritional quality.

## Materials and methods

### Model system

The experiments were performed using cultures of *Synedra* sp. (also referred to as *Ulnaria* sp. (Williams 2011); strain HS-SYN2 isolated from Feldberger Haussee, Germany), its host-specific chytrid parasitic consumer (strain SVdW-SYN-CHY1 isolated from Melzer See, DE) and *Keratella cochlearis* (isolated from Lumen Pond, The Netherlands). The chytrid has been identified as *Zygophlyctis planktonica* (Seto et al. 2020).

*Synedra* sp. is a single-celled, needle-shaped pennate diatom. The average cell length of our *Synedra* population was about 70 µm, which is larger than the reported preferred algal particle size of < 12 µm for the rotifer *Keratella* (Edmondson 1965), making *Synedra* cells likely too large to be easily ingested by *Keratella* (Frenken et al. 2016)*.* Chytrid infection of the diatom host starts with free swimming zoospores (diameter ca 2–3 µm; Seto et al. 2020) finding and attaching to a host cell. Subsequently, the zoospores encyst and form epibiontic sporangia while penetrating and digesting the host through a rhizoidal system. Within the sporangium, the next generation of zoospores is formed and finally released through rupture of the sporangium (Doggett and Porter 1996). Each infection is fatal and prohibits further reproduction of the host (Canter et al. 1992). Zoospores are small enough to be consumed by the rotifer *Keratella cochlearis* and their relatively low carbon to nutrient ratios, essential fatty acid profiles and de novo synthesis of sterols make them a valuable food source to zooplankton (Frenken et al. 2017a, b; Kagami et al. 2014; Gerphagnon et al. 2018). The tested *Keratella* species has been shown before to prey on zoospores of chytrids, even surviving and reproducing on a zoospore diet if food quantity was sufficient (Frenken et al. 2017a, b, 2020).

All organisms were grown under laboratory conditions on artificial WC medium (with addition of silicate, Guillard and Lorenzen 1972) at 18 °C and an incident light intensity of 60 µmol photons m^−2^ s^−1^ at a 16 h light: 8 h dark cycle in an incubator (MLR-350, Sanyo Electric Co., Ltd. Japan). Prior to the experiments, diatom and chytrid cultures were acclimatised to the experimental conditions in semi-continuous batch cultures for at least 2 weeks (about 10–12 generations). Diatom and chytrid cultures were grown in 1 L glass Erlenmeyers. Rotifers were cultured in 4 mL wells using 12-well plates (VWR collection, Radnor, USA) as they did not grow well in large glass Erlenmeyers and were fed ad libitum with the green alga *Chlorella sorokiniana* on WC medium under the same temperature and light conditions as the experiments. All cultures were monospecific but not axenic.

### Experimental set-up

The same experiment was performed twice, first without, and subsequently with ^15^N labelling of the host population. The natural-abundances experiment assessed the trophic position of the diatom host, chytrid and rotifer in a simple food chain based on their natural ^15^N and ^13^C isotope ratios. The N-transfer experiment quantified the transfer of nitrogen (N) from the host to the chytrid and assessed the qualitative transfer of N onwards to the rotifer. Both experiments were set up identically, except for labelling the *Synedra* culture used to start the N-transfer experiment with 10 atom% ^15^N nitrate (^15^N NaNO_3_, Sigma-Aldrich).

Each experiment consisted of three treatments (Figure ESM 1). Treatment 1 acted as control for *Synedra* population growth and population growth dilution of ^15^N labelling. Treatment 2 assessed rotifer population growth with *Synedra* as only food source and N-transfer when co-culturing *Synedra* and *Keratella*. Treatment 3 consisted of two consecutive steps, with 3a) quantifying infection dynamics and N-transfer from host to chytrids by exposing *Synedra* to chytrids, followed by 3b) co-culturing half of the infected *Synedra* cultures with *Keratella* to assess rotifer population growth with *Synedra* and chytrid zoospores as food source and N-transfer. The experiments were run in batch cultures and each treatment was replicated three times. Before starting the N-transfer experiment, the *Synedra* stock culture was ^15^N-labelled by growing it in 10 atom% ^15^N NaNO_3_ enriched WC medium for 16 h, followed by filtrating the culture over a 5 µm mesh size plankton net, resuspending it in unlabelled WC medium and starting the experiment within an hour.

The experiments were started with an average initial concentration of 650 cells mL^−1^ of *Synedra* in all three treatments, which allows several generations of population growth before nutrients can become limiting. Treatments 1 and 3a were conducted in 1 L glass Erlenmeyer flasks. Exposure to chytrids in treatment 3a was realised by adding a chytrid zoospore suspension to achieve a starting concentration of 500 zoospores mL^−1^. The zoospore suspension was prepared by gravity filtration of a heavily infected *Synedra* culture through a 5 µm mesh size plankton net. This mesh size excludes *Synedra* (average cell length 70 µm) and let zoospores pass through as their average diameter is 2–3 µm (Seto et al. 2020). At day 8 of treatment 3a, half of the volume of these *Synedra*-chytrid cultures was harvested, set to 20% infection prevalence by adding uninfected *Synedra* cells (^15^N labelled for the N-transfer experiment) and used to start treatment 3b. Treatments 2 and 3b were conducted in 24-wells (i.e. 2 × 12-well) per replicate. Rotifers were collected by pipetting and washed five times in WC medium to remove any remaining *Chlorella* cells. Each well then received 25 rotifers and either 4 mL of uninfected *Synedra* culture (Treatment 2) or 4 mL of 20% infection prevalence *Synedra-*chytrid culture (Treatment 3b). As about 900 rotifers were needed to surpass the detection limit of the elemental and isotope analysis, 24 wells constituted one replicate.

During the experiments, Erlenmeyer flasks and plates were shaken daily and redistributed randomly in the incubator to ensure averaged light conditions. Treatment 1 was run for 4 days (~ four host generations), to allow assessment of exponential population growth of the host, Treatments 2 and 3b were run for 5 days each (~ one rotifer generation), to allow incorporation of nutrients derived from feeding on zoospores or bacteria into the body tissue, and Treatment 3a was run for 8 days (~ 8 chytrid generations), to allow high infection prevalence and, therefore, sufficient zoospore biomass build up for stable isotope analysis.

### Sampling

Each replicate was sampled at start and end of the treatment. Start and end samples for treatments in glass Erlenmeyers were taken as subsamples from each replicate Erlenmeyer (Treatments 1 and 3a). To achieve sufficient biomass for stable isotope analysis, start samples for treatments in 12-well plates were taken as subsamples from the starting cultures used to fill the three sets of 12-well plates per treatment, while end samples were taken by pooling 24 wells (i.e. 2 × 12-well plates) per replicate (Treatments 2 and 3b). In treatment 3a, samples of *Synedra*-chytrids cultures were also taken at days 4, 5 and 6 to monitor population growth. *Synedra* and *Synedra*-chytrid populations of treatments 1, 2 and 3a were sampled for uninfected and infected host population counts, zoospore counts, infection prevalence and particulate organic carbon (POC) and nitrogen (PON) of two size fractions (> 5 µm: hosts + hosts with attached infections; < 5 µm: zoospores). Rotifers were sampled at the end of treatments 2 and 3b by counting the rotifer population per well, collecting and subsequently pooling rotifers of 24 wells for isotope analysis and elemental analysis. After removal of the rotifers, the *Synedra* and *Synedra*-chytrid cultures were also pooled by 24 wells and sampled for population counts, infection prevalence, POC and PON analysis. Population count samples were fixed with Lugol’s iodine solution (for diatoms, final concentration of 1% v/v) or with Glutaraldehyde (for zoospores, final concentration of 0.5% v/v) and stored at 4 °C in the dark until counting. To assess POC and PON of *Synedra*, *Synedra*-chytrid as well as pre-filtered zoospore suspensions, known volumes of each experimental unit were filtered at the start and end of the treatments over glass fiber filters (GF/F Whatman 1825-047, pore size ~ 0.7 µm). To determine the contribution of bacteria to POC and PON, we filtered the same amount of culture also over GF/C filters (Whatman 1822-024, pore size ~ 1.2 µm) assuming that most bacteria will not be retained on filters of this pore size. Rotifers were counted, collected by pipetting, washed in demi-water and transferred into a droplet of water in a tin capsule (standard size 8 × 5 mm) per replicate (i.e. 24 wells pooled). Filters and tin cups were dried at 60 °C and stored dry and dark until analysis.

### Sample analysis

#### Population growth rates and infection prevalence

Population counts of *Synedra,* chytrid-infected *Synedra* and zoospores were performed using an inverted fluorescence microscope (DMI 4000B; Leica Microsystems CMS GmbH, Mannheim, Germany) following the Utermöhl method (Lund et al. 1958). Prior to counting, the chytrid-infected samples were stained with Calcofluor White (C_40_H_44_N_12_O_10_S_2_ Fluorescent Brightener 28; Sigma-Aldrich), which binds to the chitinaceous structures of chytrid sporangia and fluoresces under UV light (Rasconi et al. 2009). As zoospores did not react with the stain, we relied on visual detection in unstained samples. For each sample, at least 400 cells or 40 fields of view (FOV) were counted which allows a ± 10% accuracy (Lund et al. 1958). Rotifer populations per well were counted using a stereomicroscope (Leica WILD MZ8, Leica Microsystems B.V., Son, The Netherlands).

#### Elemental analysis and isotopic ratios

The POC and PON samples were used to measure the elemental C and N composition as well as the *δ*^13^C and *δ*^15^N values in the different trophic levels of both, the natural abundances and the N-transfer experiment. The samples on filters were prepared for analysis according to standard methods (Teece and Fogel 2004). The samples were analysed using an elemental analyser (EA) (Flash 2000 Elemental Analyser, Thermo Scientific Bremen, Germany) coupled to an isotope-ratio mass spectrometer (Delta V Advantage Isotope Ratio Mass Spectrometer, Thermo Scientific Bremen, Germany) via a Conflo IV. Three internationally recognized standards [Arndt Schimmelmann Biogeochemical laboratories Indiana University; acetanilide, mean ± standard deviation (SD): δ^13^C = − 29.53 ± 0.01 ‰ and δ^15^N = 1.18 ± 0.02 ‰, used for calibration; urea (mean ± SD: δ^13^C = − 8.02 ± 0.05 ‰ and δ^15^N = 20.17 ± 0.06 ‰, used as standard and casein (Elemental Microanalysis Ltd; mean ± SD: δ^13^C = − 26.98 ± 0.13 ‰ and δ^15^N = 5.94 ± 0.08 ‰ used as standard] were repeatedly measured during the analysis to monitor instrument performance (i.e. 02 ‰ for ^13^C and 0.3 ‰ for ^15^N) and calculate the isotopic composition (^13^C and ^15^N) of the samples.

The isotope ratio *δ*^13^C and *δ*^15^N of the samples were expressed as the relative difference in isotope ratio between the sample and international reference standard in parts per thousand:

1 $$\delta X \left( {\permille } \right) = \left( {\frac{{{\text{R}}_{{{\text{sample}}}} }}{{ {\text{R}}_{{{\text{standard}}}} }} - 1 } \right)*1000,$$

where *δ*X is either *δ* ^13^C or *δ* ^15^N and R is the isotope ratio ^13^C/^12^C or ^15^N/^14^N in the sample and in the standard, respectively. The R_standard_ to which the samples were compared to were Vienna PeeDee Belemnite (V-PDB; 0.0112372) for carbon and air-N_2_ (AIR-N_2_; 0.003663; (Merriam et al. 2002)) for nitrogen. A positive *δ*X value indicates a higher proportion of the heavy isotopes in the sample relative to the respective standard (Hobson and Clark 1992).

### Data analysis

#### Population growth rate and infection prevalence

The counts of *Synedra,* infection and rotifers were used to calculate their net population growth rate per replicate and treatment using the following equation:

2 $${\text{Growth rate}} {\upmu } \left( {{\text{d}}^{ - 1} } \right) = \frac{{\ln \left( {X_{{2}} } \right) - \ln \left( {X_{{1}} } \right)}}{{t_{{2}} - t_{{1}} }},$$

where *X*_1_ and *X*_2_ are the cell densities at the start and end of the treatment, respectively; and *t*_2_*–t*_1_ the duration of the treatment in days. To determine the prevalence of infection, *Synedra* cells carrying living infections (*i*) and uninfected *Synedra* cells (*ui*) were counted. The prevalence of infection (*P*) was then calculated according to Rasconi et al. (2009):

3 $$P = \frac{i}{i + ui}.$$

#### C:N ratio

The carbon to nitrogen (C:N) ratio of uninfected *Synedra*, infected *Synedra*, zoospores and rotifers was calculated based on the C and N elemental composition of the samples. Comparison of the EA measurements obtained from GF/F and the GF/C filters allowed to assess the contribution of bacteria to the C:N ratio. The C:N ratio was expressed by atom, which was calculated by dividing the mass of C and N by their respective molar masses.

#### Trophic position and N-transfer

The trophic position of the chytrid in respect to its host *Synedra* and its predatory consumer *Keratella* was assessed using the δ^13^C and δ^15^N values obtained from the natural-abundances experiment. The N-transfer within our experimental food chain was quantitatively calculated for the transfer from *Synedra* to the chytrid and qualitatively assessed for the transfer from chytrids to rotifers using the δ^15^N values of the N-transfer experiment based on GF/C filters to reduce the isotope signal from bacteria as much as possible.

We expressed the N-transfer from *Synedra* to zoospores as the atom% ^15^N excess (^15^N_xs_) which is the increase of atom% ^15^N values in the zoospores relative to the atom% ^15^N values in the relevant source (i.e. *Synedra* population). The calculations are based on the equations described by Werner and Brand (2001) and Dugdale and Wilkerson (1986). By reformulating Eq. 1, R_sample_ was calculated as

4 $${\text{R}}_{{{\text{sample}}}} = \left( {\left( {\frac{{\delta {}_{ }^{15} {\text{N}}}}{1000}} \right) + 1} \right) \times {\text{R}}_{{{\text{standard}}}} ,$$

which was then used to calculate the atom% ^15^N as

5 $${\text{Atom}} \% {}_{ }^{15} {\text{N}} = \frac{{{\text{R}}_{{{\text{sample}}}} }}{{1 + {\text{R}}_{{{\text{sample}}}} }}*100,$$

and consequently, ^15^N_xs_ which is the atom% ^15^N excess in the sample over the source as

6 $${}_{ }^{15} {\text{N}}_{{{\text{xs}}}} = {\text{atom}} \% {}_{ }^{15} {\text{N}}_{{{\text{sample}}}} - {\text{atom}} \% {}_{ }^{15} {\text{N}}_{{{\text{source}}}} .$$

The relevant atom% ^15^N source for the zoospores harvested on day 8 is the atom% ^15^N of uninfected *Synedra* cells they have been growing on. Given the chytrid generation time of about 26 h (unpublished data), we estimated the atom% ^15^N value of the *Synedra* cells of day 7 while taking into account the ^15^N label dilution in the host during exponential growth by fitting an exponential model to the ^15^R data of the host control (treatment 1) (Merriam et al. 2002) as

7 $${\text{Dilution}} {}_{ }^{15} {\text{R}} \left( {{\text{d}}^{ - 1} } \right) = \frac{{{\text{ ln}}\left( {{}_{ }^{15} {\text{R}}_{2} } \right) - \ln \left( {{}_{ }^{15} {\text{R}}_{1} } \right)}}{{t_{2} - t_{1} }},$$

where ^15^R_1_ and ^15^R_2_ are the isotopic ratio ^15^N/^14^N at start and end of treatment 1, respectively; and *t*_2_*–t*_1_ the duration of the treatment in days. Using the dilution ^15^R rate, we extrapolated the ^15^R value of the *Synedra* population on day 7 to calculate the atom% ^15^N source value for Eq. 6. The N-uptake rate of the zoospores was calculated assuming a relevant assimilation time of one chytrid generation.

Finally, we assessed the ^15^N uptake rate by the chytrid on the sampling day as

8 $${}^{{15}}{\text{N uptake rate}}\left( {\mu g{\text{ N L}}^{{ - 1}} {\text{ h}}^{{ - 1}} } \right) = \frac{{{}^{{15}}{\text{N}}_{{{\text{xs}}}} \times \;\mu g\;{\text{N}}\;{\text{L}}^{{ - 1}} }}{{{\text{Generation time parasite}}}},$$

considering the uninfected part of the *Synedra* population on day 7 of treatment 3a as relevant N source to the zoospores. Hence, we multiplied the mean N content of uninfected *Synedra* cells (mean: 0.0034 $\mu$ g N cell^−1^; SD: 0.0001 $\mu$ g N) by the population density of uninfected *Synedra* (mean: 20,337 cells mL^−1^; SD: 5611 cells mL^−1^) present on day 7 in treatment 3a.

The nitrogen transfer from the zoospores to *Keratella* was qualitatively assessed by comparing the ^15^N_xs_ of rotifers in the N-transfer experiment to that of rotifers in the unlabelled controls (natural-abundances experiment) within their respective treatments (2 and 3b). While rotifers in treatment 2 were presumably starving due to the inedibility of *Synedra*, the rotifers in treatment 3b had food available in form of chytrid zoospores.

### Statistical analysis

Data were tested for normality and equality of variance prior to analyses. Outliers in the EA-IRMS analysis were detected following the criteria by Rousseeuw and Hubert (2011), resulting in the removal of five out of 114 δ^15^N values (Table ESM 2). All confidence intervals are at 95% level. 95% confidence intervals were run to determine differences in the same treatments between experiments and between comparable treatments within experiments. We compared for differences in population growth rates of the same treatments between experiments to assess whether ^15^N labelling had an influence on the growth rates; and between treatments within experiments to test for treatment effects. Specifically, we compared population growth rates of *Synedra* with and without exposure to rotifers (treatments 1 vs. 2) as well as the infection rate and zoospore production with and without exposure to rotifers (treatments 3a vs. 3b). Significant differences in population growth of rotifers exposed and non-exposed to infection were assessed with a *t *test. We also run *t* tests to determine significant difference in atom% ^15^N of rotifers fed with uninfected or infected *Synedra* (treatments 2 and 3b). Changes in C:N ratio with increasing trophic level were tested with a linear regression by numerically coding trophic levels (Post 2002) as host = 1; host-chytrid mixed cultures = 1.5; chytrids = 2, rotifers = 3. Qualitative differences in ^15^N_xs_ of rotifers exposed to infected and uninfected *Synedra* cultures were assessed with *T *tests analyses. Statistical analysis and graphing were performed in R (R-Core-Team 2012) using *RStudio (*RStudioTeam 2015).

## Results

### Population growth rate

**Fig. 2:**
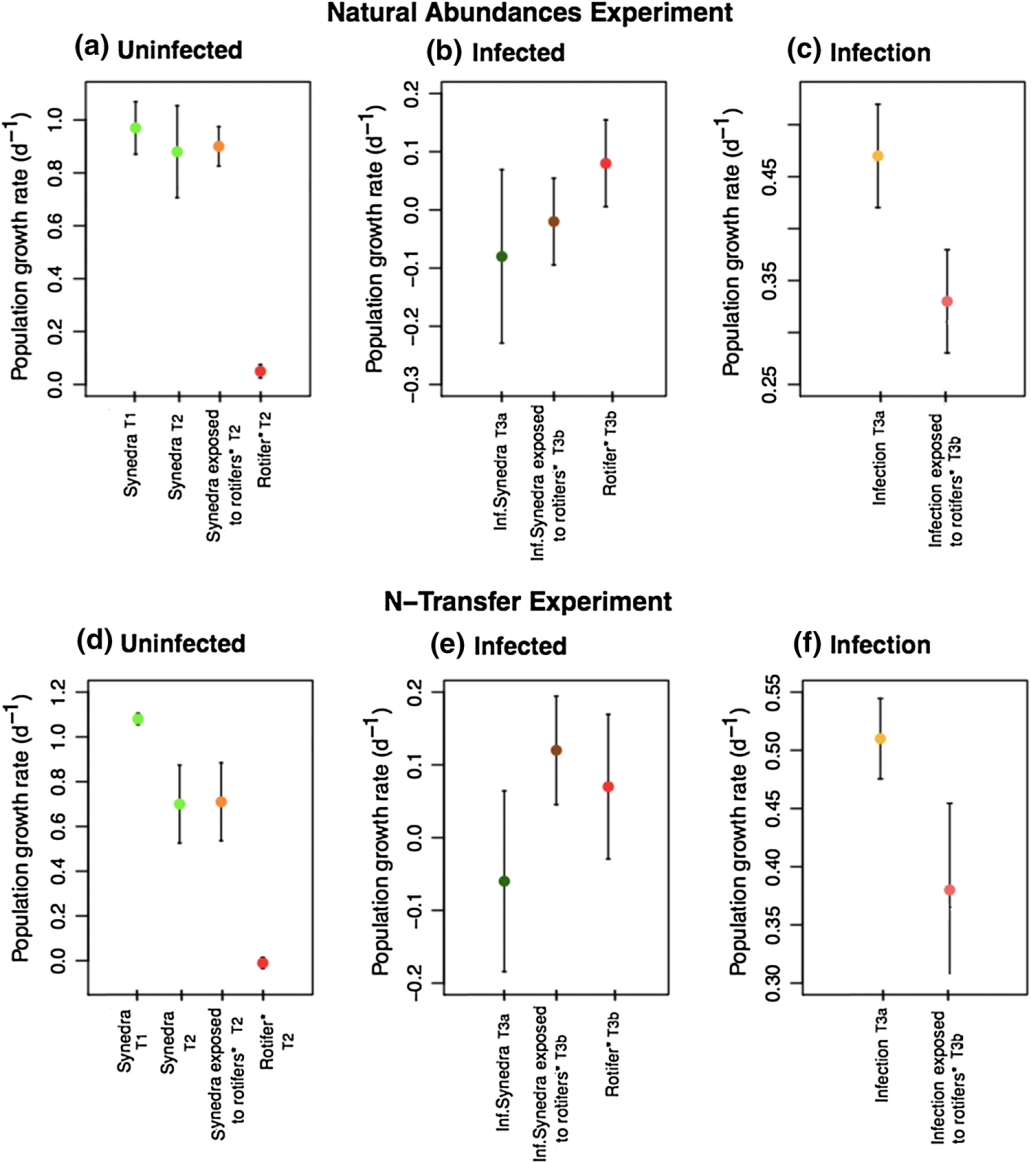
Summary of population growth rates (95% confidence interval, *N* = 3) of different species and species combinations (*Synedra*, *Synedra* exposed to rotifers, infected *Synedra*, infected *Synedra* exposed to rotifers, rotifers, infection and infection exposed to rotifers) of the three treatments (T1, T2, T3a and T3b) and the two experiments (natural-abundances and N-transfer experiments). Treatments were performed in Erlenmeyer flasks or in 12-well plates (indicated with an asterisk)

While the main focus of our study was on stable isotope results, we assessed population growth rates of uninfected and infected *Synedra*, chytrids and rotifers exposed to either uninfected or infected *Synedra* cultures to evaluate whether the overall performance of populations was similar between comparable treatments or between experiments. We expected high-population growth rates of uninfected *Synedra* alone and in co-culture with rotifers and much reduced or even negative population growth rates of infected *Synedra* due to mortality inflicted by chytrids. We also expected no population growth in rotifers exposed to uninfected *Synedra* and low but positive population growth in rotifers exposed to infected *Synedra*. The confidence interval results indicate that population growth rates per species per treatment showed some differences between experiments but responded overall similarly (Fig. 2). Population growth rate of uninfected *Synedra* in treatment 1 and 2 were clearly higher than *Synedra* growth rates in the infected treatments in both experiments (i.e. non-overlapping confidence intervals in natural-abundances and N-transfer experiment), with the highest population growth rate under labelling conditions. In treatment 2 of both experiments, the confidence intervals of population growth rates of *Synedra* exposed (glass Erlenmeyer) and non-exposed to rotifers (plastic plates) overlap, indicating a comparable population growth rate under both conditions. In treatment 3, the confidence intervals of population growth rates of infected *Synedra* (treatment 3a) and infected *Synedra* exposed to rotifers (treatment 3b) do not show much difference between the experiments. The confidence intervals of population growth rate of rotifers from treatment 2 (exposed to uninfected *Synedra*) and treatment 3b (exposed to infected *Synedra*) showed little difference between treatments or experiments; however, population growth rates of rotifers in treatment 2 were much less variable and closer to zero while the population growth rates of rotifers in treatment 3b showed wider spread and included higher values. Additionally, the *t *test results showed that in treatment 3, the population growth rate of rotifers was significantly higher than in treatment 2 (t(12) = 3.49, *p* = 0.0075). The growth rates of infection per treatment were similar in both experiments. The confidence intervals showed that population growth rates of infection (treatment 3a) are significantly higher compared to infection exposed to rotifers (treatment 3b, Fig. 2).

### Trophic position

#### Natural-abundances experiment—trophic position based on δ^15^N and δ^13^C values

**Fig. 3:**
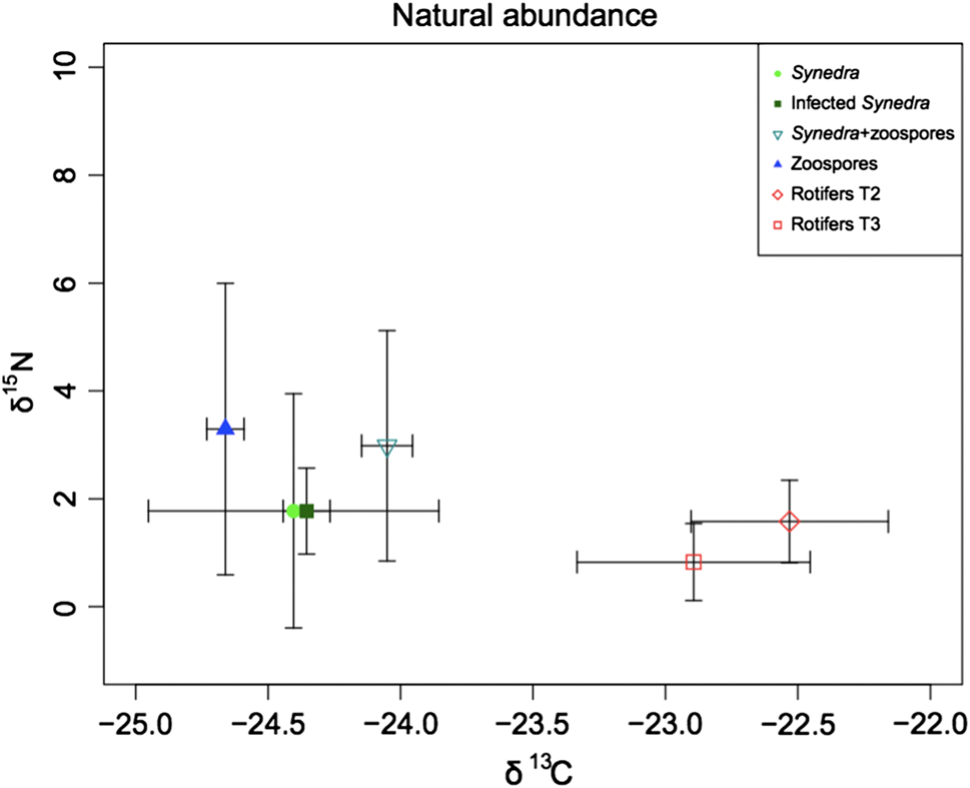
Bivariate plot of means and standard deviations of δ^13^C vs. δ^15^N values from the different predetermined trophic levels: uninfected *Synedra*, infected *Synedra*, *Synedra* with zoospore suspension, zoospores and rotifers without (treatment 2) and with zoospores of chytrids as food source (treatment 3b)

To test whether δ^15^N and δ^13^C values reflect the predetermined trophic position of diatom host, chytrids and rotifers, we calculated the means and standard deviations of the δ^15^N and δ^13^C values per species using the values of the natural-abundance experiment (Fig. 3). Rotifers were enriched in δ^13^C relative to *Synedra* and chytrid zoospores, while zoospores showed no δ^13^C change relative to their host (Fig. 3). Moreover, rotifers showed no or even negative ^15^N fractionation relative to *Synedra* or the chytrids while zoospores had variable but on average 1.5 ‰ higher δ^15^N values than *Synedra*.

### C:N ratio of trophic groups

**Fig. 4:**
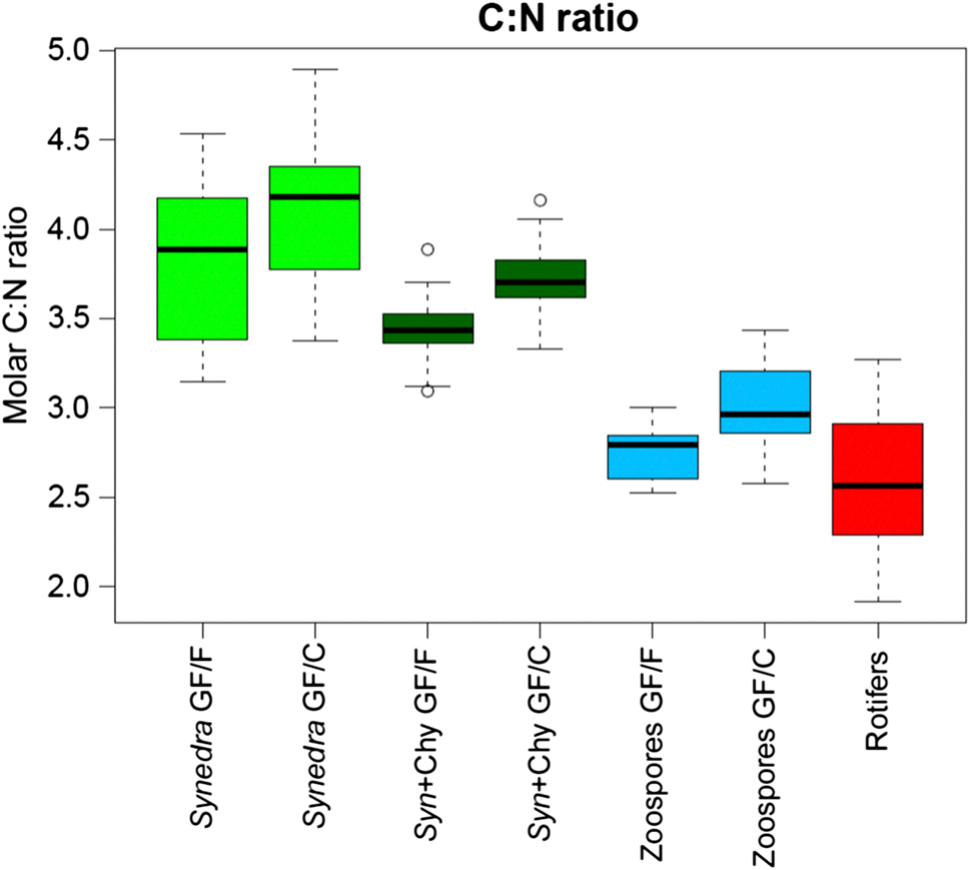
C:N ratios of the predetermined trophic levels: Representation of ratio of molar carbon to nitrogen (C:N ratio) of the filters GF/F (including bacteria, pore size ~ 0.7 μm) and the filters GF/C (excluding bacteria, pore size ~ 1.2 μm) filter measurements of *Synedra*, infected *Synedra*, zoospores and rotifers

The C:N ratio of *Synedra* samples showed a wider variation compared to that of parasitic consumers and rotifers (Fig. 4). Moreover, the C:N ratio decreased with increasing trophic level (*F*(2, 2) = 28.89, *p* = 0.032, with an *R*^2^ of 0.91). Additionally, GF/F filter-based results (i.e. filters including bacteria) showed slightly but consistently lower C:N ratios than the GF/C filters (excluding most bacteria).

### N-transfer

#### Quantitative N-transfer from *Synedra* to zoospores

The δ^15^N values obtained from the N-transfer experiment were used to quantify the nitrogen transfer in our system. The N-uptake by zoospores on day 8 was expressed as ^15^N_xs_ of zoospores relative to *Synedra*, and showed an average of 0.098 (± 0.025; Table 1). The average N-transfer rate from *Synedra* to the zoospores was 191.6 (± 57.3) µg N L^−1^ h^−1^ given a standing stock of uninfected host containing 1.8 g N L^−1^ (Table 2). Hence, at day 7 of the experiment, when the prevalence of infection was 55%, about 14% of the N content of uninfected *Synedra* was transferred to the chytrids per day.

**Table 1:** Atom% ^15^N values of zoospores and host and ^15^N_xs_ of zoospores relative to the host show parasite enrichment

Atom% ^15^N zoospores	Extrapolated atom% 15N *Synedra* on day 7 based on growth dilution of label	^15^N_xs_ of zoospores relative to *Synedra*
0.629	0.555	0.074
0.653	0.555	0.098
0.688	0.555	0.133
0.626	0.555	0.070
0.648	0.555	0.093
0.678	0.555	0.123
	Average	0.098
	SD	0.025

An exponential loss model on the *Synedra* control (treatment1) (Eq. 7) was used to calculate the growth dilution of the ^15^N label to estimate the atom% ^15^N of the host for day 7. Uptake by zoospores on day 8 was expressed as ^15^N_xs_ of zoospores relative to the *Synedra* host. Average and standard deviation (SD) of ^15^N_xs_ of zoospores relative to their host was calculated for subsequent N-uptake rate estimation

**Table 2:** N-uptake rates (µg N L^−1^ h) of the parasite were calculated using the absolute uptake (µg N L^−1^) based on the GF/F and GF/C zoospore suspension filters of day 8 (Table 2)

Type filter	µg N L^−1^ zoospores	Uptake (μg N L^−1^)	Absolute N-uptake (µg N L^−1^)	Time (h)	N-uptake rate (μg N L^−1^ h-1)
GF/F	64,772.3	0.042	2708.83	26.4	181.27
GF/C	56,173.8	0.066	3690.47	26.4	208.01
GF/F	57,354.0	0.101	5777.97	26.4	288.51
GF/C	42,039.8	0.038	1605.19	26.4	111.86
GF/F	51,199.8	0.061	3108.14	26.4	179.91
GF/C	38,729.8	0.091	3508.16	26.4	179.92
				Average	191.58
				SD	57.27

Average and standard deviations (SD) N-uptake rates by zoospores was then calculated based on the period of one generation time of the parasite

#### Qualitative N-transfer from zoospores to rotifers

The nitrogen transfer from the zoospores to the rotifers was assessed qualitatively by comparing the atom% ^15^N of rotifers in the N-transfer experiment relative to that of rotifers in the unlabelled controls (natural-abundances experiment) within their respective treatments (not exposed to chytrids: treatment 2, exposed to chytrids: treatment 3b). The difference in δ^15^N values in the N-transfer experiment samples was significant for treatment 2 (mean = 137.9; SD = 16.2) and treatment 3b (mean = 332.5; SD = 11.5) with t (1.69) = − 14.699, *p* = 0.0087 (Table 3). This result suggests that rotifers took up some *Synedra* derived N in both treatments, but that they took up relatively more *Synedra* derived N in treatment 3b.

**Table 3:** Higher ^15^N enrichment in the rotifers with zoospores as food source: atom% ^15^N of rotifers in the N-transfer experiment relative to rotifers in the (unlabelled) natural-abundances experiment per treatment suggest that uptake of *Synedra* derived N took place in both treatments 2 and 3b but there was relatively more uptake in treatment 3b

Treatment	N-transfer experiment		Natural-abundances experiment	
	δ^15^N	Atom% ^15^N	δ^15^N	Atom% ^15^N
2	126.422	0.411	0.815	0.365
2	149.312	0.419	2.343	0.366
2	NA	NA	1.590	0.366
3b	344.171	0.490	0.109	0.365
3b	321.089	0.482	0.837	0.365
3b	332.349	0.486	1.541	0.366

*NA* denotes not sufficient rotifer biomass for measurement

## Discussion

To the best of our knowledge, this study is the first that examined the relative trophic position of a phytoplankton host, its chytrid parasitic consumer and a rotifer as predatory consumer of the chytrid using their natural abundance stable of ^15^N and ^13^C isotopes and tracked the change in molar C:N ratios with trophic level. Additionally, we assessed the N-transfer from host to chytrids (quantitatively) and from chytrids to rotifers (qualitatively) using ^15^N-labelled host. Our results show no clear ^15^N enrichment in the chytrids or rotifers relative to the host, but a decreasing molar C:N ratio with increasing trophic level. Moreover, in our experimental conditions, chytrids took up about 14% of the host’s standing stock of N per day and passed N on to rotifers, showing that chytrids are well integrated in the food web.

### Population growth rate

Our results show that population growth rates were largely comparable within treatments and between experiments. The population growth rates of uninfected *Synedra* exposed and non-exposed to rotifers (treatments 1 and 2) were relatively similar, suggesting that *Synedra* growth is not affected by the presence of rotifers. Our results also show that there were only small differences in *Synedra* population growth rates between treatments performed in glass Erlenmeyer flasks (i.e. *Synedra* non-exposed to rotifers) or in plastic well plates (i.e. *Synedra* exposed to rotifers) and, therefore, we consider that *Synedra* growth rate is not affected by container type.

In the treatment 3, the infection strongly reduced the net population growth of *Synedra* overall. The net population growth rates of infection (based on prevalence of infection) as well as those of uninfected *Synedra* differed significantly between treatments 3a and 3b. In treatment 3a (no exposure to rotifers, performed in glass Erlenmeyers), prevalence of infection was higher, and the net population growth rate of uninfected *Synedra* was lower. These results align with previous studies performed by Van Donk and Ringelberg (1983), which showed that chytrid parasitism can drastically reduce a dominant diatom species population. Contrarily, in treatment 3b (exposure to rotifers, performed in plastic plates), the net population growth rate of uninfected *Synedra* was higher and the infection prevalence was lower. This result may indicate that the rotifers preyed on the zoospores and, therefore, fewer zoospores were available to infect *Synedra*. Rotifers have been shown to reduce chytrids zoospore density in another phytoplankton–host systems, although rotifer population growth showed to be limited by energetic needs and when zoospore density was insufficient, rotifer population decreased due to starvation (Frenken et al. 2017a, b, 2020).

The net population growth rate of *Keratella* in treatment 2 was lower or negative compared to the zoospore-exposed rotifer population growth in treatment 3b, suggesting that *Synedra* is not a suitable food source and as a consequence rotifer’s mortality was higher than the reproduction rate. The net population growth rates of *Keratella* in treatment 3b (0.07–0.08 d^−1^) are largely in line with population growth rates of 0.07–0.1 d^−1^ reported in the literature for *Keratella cochlearis* feeding on good quality food sources at comparable temperatures (Walz 1983; Frenken et al. 2018). However, our experiment was shorter than the reproductive cycle of the rotifers (Frenken et al. 2020). *Keratella* exhibits a low population growth rate and limited offspring compared to other species of rotifers that can reach maximum growth rates of up to 0.8 d^−1^ at 18 °C (May and Bass 1998).

### Trophic position based on natural δ^13^C and δ^15^N values

#### Natural abundance carbon isotopic composition

Estimates of δ^13^C were variable, showing little difference in δ ^13^C values between the chytrid and its host but a clear difference between rotifers and *Synedra* or chytrids. Doucett et al. (1999) proposed that parasitic consumers should be more enriched in ^13^C relative to their hosts, but the literature on host–parasitic consumer systems do not show a clear pattern of enrichment (see references in Sabadel et al. 2019). Rotifers showed an expected 1–2 ‰ enrichment in δ^13^C relative to *Synedra* and chytrid zoospores, aligning with the typical increase in δ^13^C per trophic level. Zoospores did not show a clear enrichment in δ^13^C relative to *Synedra.* The δ^13^C values were largely comparable between the different trophic levels, suggesting that rotifers, chytrids and *Synedra* were part of the same food web.

#### Natural abundance nitrogen isotopic composition

Results of δ^15^N were variable, showing a slight increase in δ^15^N in zoospores relative to *Synedra*, but no or even negative δ^15^N for rotifers relative to either *Synedra* or chytrids. Although zoospores showed a 1.5 ‰ higher mean δ^15^N value relative to *Synedra*, variability between replicates was very high. Hence, it was not possible to distinguish trophic levels in our food web based on δ^15^N values of chytrids and rotifers. Doucett et al. (1999) argued that parasitic consumers that obtain all their nutrients from the host should show the same theoretical 3.4 ‰ enrichment in δ^15^N as predators feeding on prey. This number is based on a mean of variable measurements ranging from 1.3 to 5.3 ‰ (Minagawa and Wada 1984) and is related to the nitrogen compounds that are transferred from the host to the parasite. Therefore, if the parasite does not modify the structural composition of the nitrogen compounds, it will show an isotopic composition more comparable to its food source (Chikaraishi et al. 2007). However, variability in δ^15^N values across species can be rather high, even in very closely related species, which have been on the same diet (Macko et al. 1982; Hobson and Clark 1992). In some systems, parasites do show the expected ^15^N enrichment relative to their host (Doucett et al. 1999) but in other systems, the results of stable isotope analysis have been less clear or even contradictory. For instance, parasites in fish showed ^15^N depletion relative to their host (Deudero et al. 2002). Other studies (Deudero et al. 2002; Lafferty et al. 2008) showed that parasites can be depleted, enriched or have similar δ^15^N as compared to the host depending on the species of parasite and the host. In addition, the same parasite species in different hosts or different parasites species on the same host might have very different δ^15^N values (Lafferty et al. 2008). The reason for this inconsistency in the stable isotope response of parasites is still unresolved. Part of this variation in trophic discrimination factors of parasites may come from selective feeding on specific host substrates that differ in isotopic signature resulting in different enrichment patterns in the parasite (Deudero et al. 2002; Sabadel et al. 2019). In our host–parasite system, the chytrid infection kills the host and leaves an empty host cell without apparent residual cell contents. However, it remains unclear whether the chytrid actually consumes all cell contents of the diatom or whether there is leakage of specific compounds during the infection that could explain our result in a slight, but variable ^15^N enrichment in the parasite.

The rotifers also did not show enrichment in ^15^N relative to *Synedra* or the chytrid. Contrarily, they seemed to be depleted in ^15^N compared to the zoospores. The δ^15^N values obtained from the rotifers from treatment 2 (rotifers exposed to *Synedra*) were similar to those of uninfected *Synedra*. Low or even negative net population growth rates of rotifers in treatment 2 suggest that rotifers were starving. Moreover, pre-experiments showed that the population of *Keratella* exposed to *Synedra* as only food source collapsed and died within 9 days. This would agree with other studies that suggested that the size of *Synedra* cells is too large to be ingested by *Keratella* (Frenken et al. 2016). However, under starvation, an increase in δ^15^N would be expected (Gaye-Siessegger et al. 2007) which we do not observe in our results. Rotifers that were exposed to zoospores (treatment 3b) also showed ^15^N depletion compared to the δ^15^N values of *Synedra* and the zoospores, even though the population growth rate of rotifers in this treatment was comparable to growth rates under good food conditions (Frenken et al. 2018). It is also unlikely that the rotifer δ^15^N values are a legacy of the culture maintenance conditions as the food source (*Chlorella sorokiniana*) showed a higher δ^15^N (mean = 3.611; SD = 0.425, data not shown). However, we cannot fully exclude that the low δ^15^N of rotifers might reflect a change in their internal use of N due to starvation. How rotifers adjust their physiology when they undergo starvation conditions is not well understood (Kirk 1997). The protein content of rotifers can be very changeable and it is strongly related to their diet and cultivation conditions (Makridis and Olsen 1999). In other rotifer species, biomass, nitrogen and amino acid content decrease exponentially with time when they experience deprivation of food quantity or quality, especially at high temperatures (20–28 °C) (Makridis and Olsen 1999). These internal changes could explain why the rotifers did not show enrichment in δ^15^N compared to their food source.

### Carbon to nitrogen ratio

In line with expectations, *Synedra* showed the highest and most variable C:N ratio, while zoospores and rotifers displayed a narrower range with lower C:N ratios. Elemental composition of C, N and P differ between consumers and primary producers and these stoichiometric differences can be used as indicators of ecological processes such as nutrient cycling and trophic structure (Frost and Elser 2002).

Grazers typically have higher nutritional demands with lower and less flexible C-to-nutrient ratios than primary producers (Sterner and Elser 2002). Our results support these findings, where the nitrogen content relative to carbon of zoospores and rotifers is significantly higher (i.e. lower C:N ratio) compared to that in *Synedra*. Consequently, chytrids may stoichiometrically upgrade the food for rotifers, which in turn may do so for higher trophic levels. We expected that the C:N ratio would decrease with higher trophic position as carbon is used in heterotrophic metabolisms while at the same time nutrients are becoming concentrated in higher trophic levels (Sterner et al. 1998; Boersma et al. 2008). We note that *Synedra* is relatively N rich in comparison to the Redfield C:N ratio of 6.6, suggesting that the diatoms were not N limited during the experiment. Also, the chytrid is relatively N-rich in comparison to literature values for pathotroph fungi (Zhang and Elser 2017) or aquatic saprophytic fungi (Danger et al. 2016).

The zoospore elemental composition seemed closer to that of rotifers while the infected *Synedra* values were more similar to uninfected *Synedra.* Herbivorous consumers generally have higher nutrient demands than their plant food source and this higher requirement is reflected in their lower C:P and C:N ratios (Van de Waal et al. 2010; Hessen et al. 2013). Paseka and Grunberg (2019) showed that parasite C:N ratios can differ depending on life-cycle stage with lower C:N ratios during active reproduction. In line with earlier work (Frenken et al. 2017b, 2018), our findings show that zoospores appear to be efficient in transferring nutrients from their host to rotifers. This process can work in two ways: first by direct transfer of *Synedra* biomass to rotifers and upgrading the quality of the food, e.g. lower C:N ratios, for the rotifers. Second, the presence of chytrids might be linked to higher amounts of bacteria (personal observation), potentially through leakage of organic matter from the infected host. If we compare the GF/F filters (including bacteria) and the GF/C (excluding most bacteria), filters with more bacteria seem to show a lower C:N ratio, suggesting that the bacteria may also improve the quality of the food.

### N-transfer based on ^15^N labelling experiment

Based on tracing the ^15^N label from host to the chytrid, we showed that ∼14% of the N content of *Synedra* was transferred to the parasitic consumers per day when the prevalence of infection was 55%. Although there are to date no studies that quantify the transfer of nutrients from phytoplankton to chytrids, Grami et al. (2011) developed a model to predict changes in carbon transport in a natural lake community due to chytrid parasitism on phytoplankton. According to this model, around 22% of the carbon of the phytoplankton is transferred to chytrid sporangia, 18% to the zoospores and 12.5% to zooplankton, resulting in less sedimentation loss of phytoplankton carbon and longer carbon path lengths. Our N-flux estimates suggest a comparable percentage of N transferred from hosts to zoospores and supports the hypothesis that chytrid parasitism can affect nutrient flows in aquatic food webs.

We assessed qualitatively the transfer of N from zoospores to rotifers as expressed in ^15^N_xs_. Rotifers exposed to infected *Synedra* showed higher atom% ^15^N compared to rotifers exposed to *Synedra* only, indicating an overall larger flux of N from resource to the rotifers when zoospores were available as food source. However, the rotifers experiencing starvation (*Synedra* only treatment) still showed a low amount of ^15^N labelling, suggesting that the rotifers were obtaining some ^15^N either directly from excreted extracellular material of *Synedra* or from bacteria feeding on these *Synedra* exudates. Rotifers exposed to chytrid-infected *Synedra* culture showed relatively more labelling compared to the *Synedra* only treatment, suggesting that these rotifers were obtaining more diatom host-derived nutrients. The mechanism for this is presumably through the direct intake of labelled zoospores as infected *Synedra* cells did not become more edible for the rotifer as the host silica frustules remain intact during and after infection. However, increased organic matter, including nitrogen, recycling through heterotrophic bacteria as a result of the parasitic infection (Senga et al. 2018) and, therefore, indirect labelling of the rotifers cannot be entirely excluded. Nevertheless, the labelling signal of the rotifers was very low when compared to zoospores and *Synedra* and we cannot exclude that rotifers were starving also in presence of zoospores. Rotifers can ingest ~ 5–10 µL d^−1^ (Rothhaupt 1995) and the density of zoospores at the end of the experiment was ~ 20 zoospores µL^−1^. The density of zoospores might have been too low for the rotifers to thrive despite zoospores being a good food quality source (Gleason et al. 2008).

## Conclusions

Our results of the natural-abundances experiment confirm current knowledge that chytrids completely depend on their host as food source as their δ^13^C was indistinguishable from their host. However, the expected ^15^N enrichment relative to their host was absent. Hence, assessment of the trophic position of the chytrid based on measuring stable isotopes of whole tissue (“bulk”) samples remains inconclusive. The potential of using stable isotopes for assessing the food web position of rotifers remained unresolved. Despite clear ^13^C enrichment of 1 to 2 ‰ relative to the food source as expected, we observed no ^15^N enrichment relative to host and parasitic consumer. While we cannot exclude starvation despite the presence of edible zoospores, net population growth rate in the zoospore-exposed treatment seemed not to support a severe starvation scenario. Our C:N ratio results showed that zoospores are relatively N rich and have a relatively similar C:N ratio as rotifers, suggesting that they are a high-quality food source for zooplankton. We could also confirm substantial transfer of N from host to chytrids and show some evidence that the host N was also transferred via chytrid infections to zooplankton, supporting the mycoloop concept and suggesting a role for chytrids in rerouting N flows from inedible producers to predatory consumers.

Future assessments of the trophic position of parasites may profit from using more informative stable isotope methods such as the compound-specific isotope analysis of amino acids, which allows much higher accuracy in trophic position estimation (Chikaraishi et al. 2007; Steffan et al. 2013; Sabadel et al. 2016). Furthermore, linking information on elemental ratios of organisms to their stable isotope signatures may help to assess the parasite trophic position, and understand the underlying mechanism of elemental imbalances between hosts, parasitic consumers and predatory consumers of parasitic consumers.

## Electronic supplementary material

Below is the link to the electronic supplementary material.

Supplementary file 1 (PDF 115 kb)
