# Supplementary material for: Trophic position, elemental ratios and nitrogen transfer in a planktonic host–parasite–consumer food chain including a fungal parasite
Source: Oecologia. 2020 Aug 17;194(4):541–54. doi: 10.1007/s00442-020-04721-w (PMC7683484; doi:10.1007/s00442-020-04721-w)
Supplement: Supplementary file 1 — (PDF 115 kb) [file 442_2020_4721_MOESM1_ESM.pdf]

Trophic position, elemental ratios and nitrogen transfer in a planktonic host-parasite-consumer food chain including a fungal parasite

Figure ESM1

Fig. ESM1. Scheme of the three treatments performed in each experiment. Treatment 1 was a control of *Synedra* growth and labelling dilution; Treatment 2 tested if rotifers feed on *Synedra* and if N-transfer could be observed; Treatment 3 a) tested N- transfer from *Synedra* to chytrids 3b) assessed if rotifers feed on zoospores of chytrids and if N-transfer could be observed. Run twice, one labelled and one unlabelled

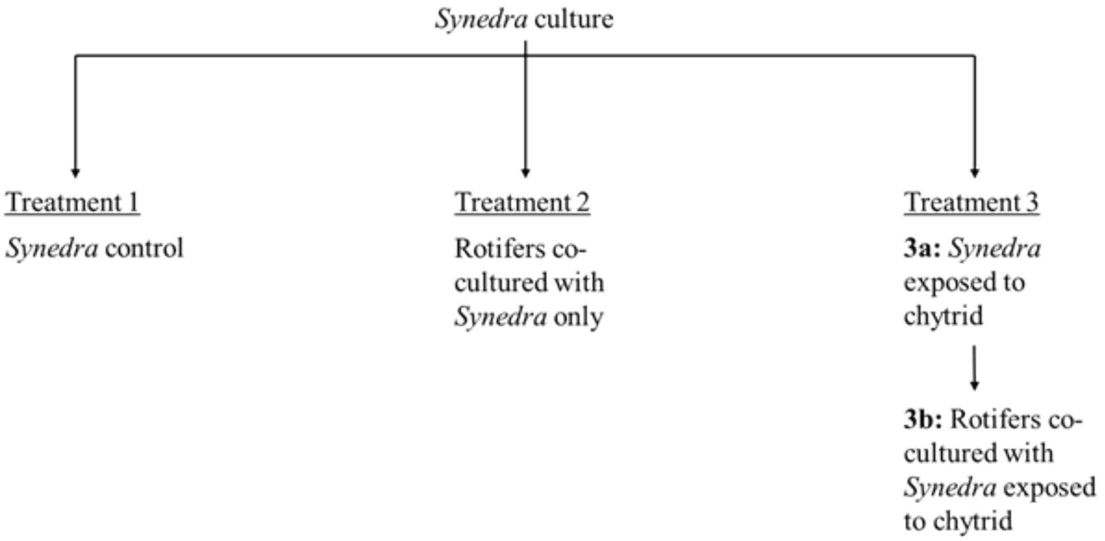

Table ESM2

Table ESM2. $\delta^{15}N$ values obtained from the IRMS analysis that were rejected according to the methods developed by Rousseeuw & Hubert (2011)

Set-up	Date	Treatment	Organism	Type of filter	$\delta^{15}N$	Reason to be excluded
N-Transfer Experiment	10/7/17	T3b	Infected Synedra plates	GF/C	13.72	SD more than 3 times than rest of the values
N-Transfer Experiment	5/7/17	T2	Synedra from rotifers	GF/C	2.27	IRMS measurement error
N-Transfer Experiment	5/7/17	T2	Synedra from rotifers	GF/C	-4.29	IRMS measurement error
N-Transfer Experiment	22/6/17	T3b	Synedra +zoospores	GF/F	-1.02	IRMS measurement error
N-Transfer Experiment	29/6/17	T3b	Zoospores	GF/C	-6.38	IRMS measurement error

Reference

Rousseeuw PJ, Hubert M (2011) Robust statistics for outlier detection. Wiley Interdisciplinary Reviews: Data Mining Knowledge Discovery 1:73-79
